# Evaluation of Optogenetic Electrophysiology Tools in Human Stem Cell-Derived Cardiomyocytes

**DOI:** 10.3389/fphys.2017.00884

**Published:** 2017-11-02

**Authors:** Susann Björk, Elina A. Ojala, Tommy Nordström, Antti Ahola, Mikko Liljeström, Jari Hyttinen, Esko Kankuri, Eero Mervaala

**Affiliations:** ^1^Department of Pharmacology, Faculty of Medicine, University of Helsinki, Helsinki, Finland; ^2^Department of Physiology, Faculty of Medicine, University of Helsinki, Helsinki, Finland; ^3^BioMediTech Institute and Faculty of Biomedical Sciences and Engineering, Tampere University of Technology, Tampere, Finland; ^4^Department of Anatomy, Faculty of Medicine and HiLIFE, University of Helsinki, Helsinki, Finland

**Keywords:** optogenetics, human iPSC-derived cardiomyocytes, optical action potential, contractile motion, hERG, cardiac electrophysiology, arrhythmia, safety pharmacology

## Abstract

Current cardiac drug safety assessments focus on hERG channel block and QT prolongation for evaluating arrhythmic risks, whereas the optogenetic approach focuses on the action potential (AP) waveform generated by a monolayer of human cardiomyocytes beating synchronously, thus assessing the contribution of several ion channels on the overall drug effect. This novel tool provides arrhythmogenic sensitizing by light-induced pacing in combination with non-invasive, all-optical measurements of cardiomyocyte APs and will improve assessment of drug-induced electrophysiological aberrancies. With the help of patch clamp electrophysiology measurements, we aimed to investigate whether the optogenetic modifications alter human cardiomyocytes' electrophysiology and how well the optogenetic analyses perform against this gold standard. Patch clamp electrophysiology measurements of non-transduced stem cell-derived cardiomyocytes compared to cells expressing the commercially available optogenetic constructs Optopatch and CaViar revealed no significant changes in action potential duration (APD) parameters. Thus, inserting the optogenetic constructs into cardiomyocytes does not significantly affect the cardiomyocyte's electrophysiological properties. When comparing the two methods against each other (patch clamp vs. optogenetic imaging) we found no significant differences in APD parameters for the Optopatch transduced cells, whereas the CaViar transduced cells exhibited modest increases in APD-values measured with optogenetic imaging. Thus, to broaden the screen, we combined optogenetic measurements of membrane potential and calcium transients with contractile motion measured by video motion tracking. Furthermore, to assess how optogenetic measurements can predict changes in membrane potential, or early afterdepolarizations (EADs), cells were exposed to cumulating doses of E-4031, a hERG potassium channel blocker, and drug effects were measured at both spontaneous and paced beating rates (1, 2 Hz). Cumulating doses of E-4031 produced prolonged APDs, followed by EADs and drug-induced quiescence. These observations were corroborated by patch clamp and contractility measurements. Similar responses, although more modest were seen with the I_Ks_ potassium channel blocker JNJ-303. In conclusion, optogenetic measurements of AP waveforms combined with optical pacing compare well with the patch clamp gold standard. Combined with video motion contractile measurements, optogenetic imaging provides an appealing alternative for electrophysiological screening of human cardiomyocyte responses in pharmacological efficacy and safety testings.

## Introduction

Present cardiac safety assessments focus on the *in vitro* block of the human rapid component of the delayed inward rectifier I_Kr_ (hERG) channel, combined with *in vivo* QT prolongation for evaluating the arrhythmic risks of novel drug candidates in preclinical development. Block of the hERG channel delays cardiac repolarization, prolonging the action potential duration (APD) and the QT interval on ECG, and potentially increases the risk for the development of the cardiac arrhythmia Torsades de Pointes (TdP) (Sanguinetti et al., 1995; Redfern et al., 2003; Gintant et al., 2016). However, although these assays have been effective in preventing drugs that induce TdP proarrhythmia from entering the market, it has been recognized that a drug's proarrhythmic effect often is shaped by its action on multiple ion channels (Mirams et al., 2011). The lack of specificity of the hERG assay therefore often leads to unwarranted attrition of drugs, which is costly for the pharmaceutical industry. A more focused approach to address and eliminate cardiovascular toxicity early in development has thus been proposed by the Comprehensive *in vitro* Proarrhythmia Assay (CIPA) initiative (Gintant et al., 2016). CIPA proposes a multimodal approach of cardiac safety screening based on the integrated effects of drugs on the multiple cardiac ion channels that define cardiac excitability and repolarization and that play a role in delayed ventricular repolarization. Reconstructions of the drug effects are evaluated *in silico* on a computationally reconstructed human ventricular cardiomyocyte action potential (AP) (Cavero and Holzgrefe, 2014; Fermini et al., 2016; Gintant et al., 2016; Page et al., 2016). Finally, predicted effects are verified with electrophysiological experiments in human induced pluripotent stem cell-derived cardiomyocytes (hiPSC-CM).

Numerous methodology development studies have appeared which have tried to assess the criteria set up by CIPA. Optical, non-invasive measurements of AP parameters performed in hiPSC-CM has been associated with great potential over the current gold standard, patch clamping, since it focuses on the AP waveform from multiple cells beating synchronously and thus assesses the contribution of several ion channels on the overall drug effect (Entcheva, 2013; Ambrosi and Entcheva, 2014; Chang Liao et al., 2015; Dempsey et al., 2016; Klimas et al., 2016). Optogenetics utilizes light sensitive proteins (microbial opsins), that are genetically encoded and expressed on the cardiomyocyte plasma membrane, where they function as optical actuators or sensors, which enables all-optical shaping of the AP. Hochbaum and colleagues developed several optogenetic constructs, of which Optopatch2 utilizes a modified version of the channelrhodopsin cation channel (CheRiff) that in response to blue light at 488 nm depolarizes the cardiomyocyte, enabling pacing of cardiomyocytes at elevated beating rates. By combining this optogenetic actuator with the genetically encoded voltage indicator QuasAr2, a modified, non-pumping version of the protein pump Archaerhodopsin3, which in response to red light at 640 nm generates an optical signal that is proportional to the membrane potential, all optical electrophysiological experiments were demonstrated in neuronal cells (Hochbaum et al., 2014), and later in hiPSC-CMs (Dempsey et al., 2016). Another construct CaViar, based on the genetically encoded voltage sensor Arch(D95N) combined with the genetically encoded calcium sensor, GCaMP5f, was developed to allow for simultaneous AP dynamics and intracellular calcium transient determinations (Hou et al., 2014).

Traditional electrophysiological methods, such as calcium imaging, measures the ionic functions which regulate the contractile movement of the cells. However, these measurements do not directly quantify the biomechanics of the cell. Different video-based block matching methods have been developed to non-invasively measure the contractile movement in cardiomyocytes and the results on cellular biomechanics have been linked to clinical findings (Kiviaho et al., 2015; Laurila et al., 2016). Since contractile cardiotoxicity also is a safety concern, combining optogenetic electrophysiology experiments with contractile measurements would therefore bring added value to safety screens. Furthermore, the arrhythmogenic sensitizing by light-driven pacing in combination with optical measurements of cardiomyocyte APs is essential to detect toxic drug effects evident only under elevated beat rates. Due to the stringent and meticulous requirements of cardiac safety testing it is of utmost importance that these novel tools are studied in detail to understand how the optogenetic modifications possibly alter human cardiomyocytes' electrophysiology. The purpose of this study was to characterize the optogenetic constructs (Optopatch and CaViar) against non-transduced cardiomyocytes, and more importantly, to compare how well-optogenetic analyses perform against the gold standard, patch clamp electrophysiology.

## Materials and methods

### Cell culture

Human induced pluripotent stem cell-derived cardiomyocytes (hiPSC-CMs), Cor.4U®, were acquired from Axiogenesis Inc. (Germany). These spontaneously beating cells represent a mixture of atrial, nodal and ventricular cardiomyocytes, with 60% being of the ventricular type. Cor.4U® hiPSC-CMs were delivered as fresh cells in T25-flasks (Nunc^©^, Thermo Fisher Scientific) and kept in an incubator (5% CO_2_, 37°C) and fed daily with Cor.4U® complete culture medium (Axiogenesis Inc.) supplemented with 1X Antibiotic-Antimycotic (Thermo Fisher Scientific) to prevent bacterial and fungal infection. For further passaging T25 flasks were pre-coated with 10 μg/ml fibronectin (Sigma) in PBS (with Ca^2+^ and Mg^2+^, Gibco®, Thermo Fisher Scientific) for 3 h at 37 or at 4°C o/n and the solution was removed shortly before plating the cells. For passaging, cells were detached using Accumax (Millipore) according to the manufacturer's instructions. The cells were collected by centrifugation (200 g, 3 min), the supernatant was removed and the cell pellet was gently re-suspended in the culture medium. Viable cells were counted using the trypan blue exclusion method and the cell density calculated according to viable cells. After plating, the cells were kept in the cell culture hood for 15 min to ensure that the cells settled evenly.

### Lentiviral transduction of cardiomyocytes

To express light-gated voltage sensors and actuators on the plasma membrane of the hiPSC-CMs, the cells were transduced with lentiviral vectors bearing the constructs of interest. CaViar, pJMK019 (Addgene plasmid # 42168) and CMV-Optopatch2_FCK, pMOS001 (Addgene plasmid # 62984) were gifts from Adam Cohen, acquired through the non-profit plasmid repository Addgene. The production of lentiviral particles, lentiviral titer determinations and replication competent virus (RCV) tests were purchased from the Biomedicum Virus Core Unit in the Faculty of Medicine, University of Helsinki. For the toxicological testing of lentiviral particles, four different amounts of lentivirus stock were tested for both optogenetic constructs (0.25, 0.50, 0.75, and 1.00 pg/cell for CaViar; 0.50, 0.75, 1.00, and 1.25 pg/cell for Optopatch, as determined by the p24 capsid protein concentration), where final concentrations used are underlined. For the transduction procedure, the lentiviral stock was diluted 1:2 in serum-free BMCC medium (Axiogenesis) supplemented with polybrene (4 μg/ml final concentration) to assist the penetration of the viruses through the cell membrane. The cells were incubated for 6–7 h with the lentivirus mix and then washed with PBS to remove excess virus. The lentiviral transduction was confirmed by CheRiff-tagged GFP or GCaMP5f introduced in the transduced cells, imaged by an EVOS® FL Imaging System. After the lentiviral transduction, the cells were washed with 3x PBS and supplemented with fresh culture medium daily. 24 h after the transduction procedure the cells were screened for cytotoxicity and cytolysis using an absorbance-based lactate dehydrogenase (LDH) release assay (Pierce, Thermo Fisher Scientific). In order to get rid of the replicative virus prior to patch clamp measurements and optogenetic imaging, the transduced cells were passaged for two times during a time period of 2–3 weeks in a BSL2 safety level laboratory. Passaging was done as described above and cells were re-plated in fibronectin-coated T25 flasks.

### Patch clamp electrophysiological measurements

Whole-cell recordings were performed using an EPC 9/2 double patch clamp amplifier and pulse v 8.80 software (HEKA Elektronik, Lambrecht, Germany). For current clamp recordings, non-transduced control hiPSC-CMs and hiPSC-CMs expressing the optogenetic constructs were plated as sub-confluent monolayer in fibronectin-coated petri dishes (30 mm, Nunc), which were placed on an inverted microscope (Olympus IX71) and visualized using an AxioCam HRM digital camera (AxioVision 4.6 software). For the recordings cells were perfused with a bathing solution composed of 143 mM NaCl, 4 mM KCl, 1.2 mM MgCl_2_, 1.8 mM CaCl_2_, 5 mM D-glucose, and 10 mM HEPES (pH 7.4 NaOH). The internal pipette solution contained 122 mM K^+^-Gluconate, 30 mM KCl, 1 mM MgCl_2_, 5 mM HEPES (pH 7.2, KOH). The microelectrodes were pulled from borosilicate glass (outer diameter 1.5 mm) on a two-stage pipette puller (PC-10, Narishige) and heat polished with a Micro Forge MF-90 heater (Narishige). The resistance of the pipettes used in the experiments were 2.5–3.5 MΩ. Membrane capacitance and series resistance were compensated electronically. The HEKA amplifier was set to current clamp at zero applied current, and spontaneous APs were recorded for 20 s in each data sweep. The cells were superfused with the bathing solution at a rate of 1.0 ml/min. All experiments were done at 37°C by using a TC-344B Dual automatic temperature controller (Warner). To minimize the volume in the petri dish, a petri dish insert was used (Bioscience Tools). Action potentials were digitized at 10 kHz and low-pass filtered at 3 kHz.

### Preparation of cells for optogenetic measurements

For the optogenetic imaging, transduced hiPSC-CMs were plated as confluent monolayer on Geltrex (Gibco®, Thermo Fisher Scientific)-coated glass-bottom dishes (10 mm Ø, P35G-1.5-10-C, MatTek), by seeding 90,000 cells per dish. Geltrex was pre-incubated on the glass-bottom dishes at 37°C for 1 h and removed shortly before plating the cells. After plating, cells were left in the cell culture hood for 15 min to ensure an even monolayer of the cells. Cells were cultured on glass-bottom dishes for 1 week to ensure full integration of the beating monolayer. Just before the optogenetic imaging the culture medium was exchanged for imaging buffer, which was identical to the patch clamp bathing solution. Separate dishes were utilized for spontaneous beating, 1 and 2 Hz pacing (three dishes for each condition).

### Drug dilutions

Dried powders of E-4031 and JNJ-303 (Tocris) were dissolved in DMSO to make a stock concentration of 10 mM. Compounds were solubilized by vortexing the stock solution at RT and stock solutions were stored at −20°C until use. The drug dilutions were prepared fresh at the day of the experiment from stocks in imaging buffer and kept at 37°C in 5% CO_2_. For optogenetic imaging, the addition of drugs started from a blank (fresh imaging buffer) to check proper beating of the monolayer, followed by vehicle and drug doses. The entire volume (2 ml) in the dish was exchanged at each drug dose, as we noted that the cells needed fresh buffer at regular intervals for proper beating. A delay of ~1 min before imaging was allowed in order for the drug to take effect. The vehicle DMSO concentrations were 0.001% (v/v) for E-4031 series and 0.03% (v/v) for JNJ-303 series. The final concentrations for E-4031 were 3, 10, 30, and 100 nM and the final concentrations for JNJ-303 were 0.03, 0.1, 0.3, 1, 3, and 10 μM. For E-4031 patch clamp measurements drugs were diluted in bathing solution and administered through perfusion.

### Optogenetic measurement setup

The optogenetic imaging platform was designed for fast photo manipulation and analysis of live cells. The platform included an environmental chamber (5% CO_2_, 37°C, EMBL), a fully motorized inverted wide field epifluorescence microscope (Nikon Eclipse Ti-E equipped with a Nikon IR-based Perfect Focus System, PFS). Pulses (10 ms) of blue laser illumination (Argon, λ = 488 nm, 17 mW/mm^2^) were used to pace CheRiff at 1 or 2 Hz frequencies and a red laser λ = 647 nm, E_f_ = 550 mW/mm^2^ excited fluorescence of QuasAr2. For CaViar imaging, the laser lines were used in combination with a beam splitter (Hamamatsu Gemini) to allow simultaneous use of the two separate illumination sources (λ = 647 nm, E_f_ = 550 mW/mm^2^ to excite fluorescence of ArchD95N and λ = 488 nm, E_f_ = 3 mW/mm^2^ for GCaMP5f). Fluorescence was collected via a 60× oil immersion objective (PlanApo VC) with a numerical aperture (NA) of 1.4. Illumination was limited to the ocular field of view (FOV, 22 mm) of the Nikon Ti-E inverted microscope by adjusting the field stop. The illuminated area was calculated from the FOV and the objective magnification (giving a surface area of 0.106 mm^2^). Optical power was measured on the microscope sample plane with an EXFO X-Cite XR2100 power meter. Laser illumination at 488 nm was measured with the acousto-optic tunable filter (AOTF) set to 100% transmission (giving a power reading of 1.8 mW). At 647 nm the laser output was set to 100 mW and the AOTF transmission was limited to 50% to avoid saturation (giving a power reading of 9.7 mW). The actual laser power used for imaging at 647 nm (laser output 300 mW, AOTF 100%) was calculated assuming a linear response (resulting in a power of 58.2 mW). The software for the platform operation was NIS-Elements advanced research v. 4.2 with 6D image acquisition module. Signals were recorded with an Andor iXon3 897 back-illuminated EMCCD camera (512 × 512 px) or Andor iXon+ 885 EMCCD camera (1,004 × 1,002 px) for CaViar. Imaging was conducted at a framerate of 50 frames per second. The raw imaging data from optogenetic imaging was recorded as image sequences, from which the total intensity signal was exported to MS Excel in numerical format. The raw data trace was acquired as an average signal from the cells in the whole FOV. To calculate averages for each condition or drug concentration, image sequences from six FOVs were recorded.

### Automated data processing and curve analysis

For the automated processing of optogenetic raw data traces and the analysis of key features of cardiac electrophysiology, we developed the cPot Cardiac Action Potential Calculator software, written in MATLAB. With cPot, all raw data traces from optogenetic imaging were normalized by fitting the acquired signal to an exponential function. Then, peaks with larger than a selected threshold (10% of maximum amplitude) were detected in the normalized signal. The detected peak time points and their respective signal values were then used to determine the AP parameters and other key features. The key features analyzed and reported in this study were APD at 90, 50, and 30% repolarization (APD90, APD50, and APD30, s, respectively), beat to beat interval (s), frequency (Hz), maximum signal level of the peak, i.e., amplitude (ΔF/F for optogenetics, mV for patch clamp) and minimum signal level between peaks (MDP). Respective percentage levels for APDs were determined so that 100% was the overall change in signal from Peak Height to the following MDP. Patch clamp data was analyzed with cPot in the same way, but without normalization since the baseline in patch clamp measurements is steady. Optogenetic calcium traces for contraction analysis were normalized by fitting the acquired signal to an exponential function.

### Simultaneous contraction analysis of video microscopy

The contractile movement of the cardiomyocytes was analyzed from Optopatch and CaViar video microscopy sets using a semi-automatic CellVisus tool (Ahola et al., 2014). It uses particle image velocimetry based on minimum quadratic difference to determine velocity vector fields between consecutive video frames. Directional motion velocity signals are calculated from AP video data by using an estimated beating focus point as a reference. Contraction signals are generated from these motion velocity signals by integrating with respect to time and fitting the signal on a spline for baseline correction. Contraction amplitude was normalized to comply with the AP and the calcium transient for illustration. Here, we analyzed the motion from AP measurement in both Optopatch and CaViar microscopy from 10 image sequences each.

For signal characterization, calcium transient duration (CTD) parameters at different amplitude levels were calculated. Calcium transient durations (CTD) parameters CTD90, CTD50, and CTD30 were calculated by determining percentage values so that 100% was the overall change in signal from Peak Height to the following MDP. For contraction movement, we calculated the contraction time and relaxation time, as well as total contraction duration (CD) parameters CD90, CD50, CD30 defined by the beginning of the contraction and the end of relaxation movement. Further, we measured the time difference between the AP, calcium transient and contraction signal peaks from the same region of interest. The effect of E-4031 to contraction was measured by analyzing in total 92 image sequences for vehicle and 3, 10, 30 and 100 nM drug concentrations. In addition to the CD parameters listed above, average motion magnitude was measured.

### Statistical testing

The APD-values were beat rate adjusted, so that beating intervals were corrected to 60 bpm by Fridericia's correction formula (based on the cube-root of beating interval). Statistical comparisons were done either with a Student's two-sample *t*-test, or for cumulating drug responses with a one-way ANOVA with Dunnett's test for statistical significance. Significant *p*-values were ^*^p < 0.05, ^**^p < 0.01, ^***^p < 0.001.

## Results

### Lentiviral transduction of hiPSC-CMs with optogenetic constructs exhibit no significant side effect on the electrophysiological properties of cardiomyocytes

**Figure 1:**
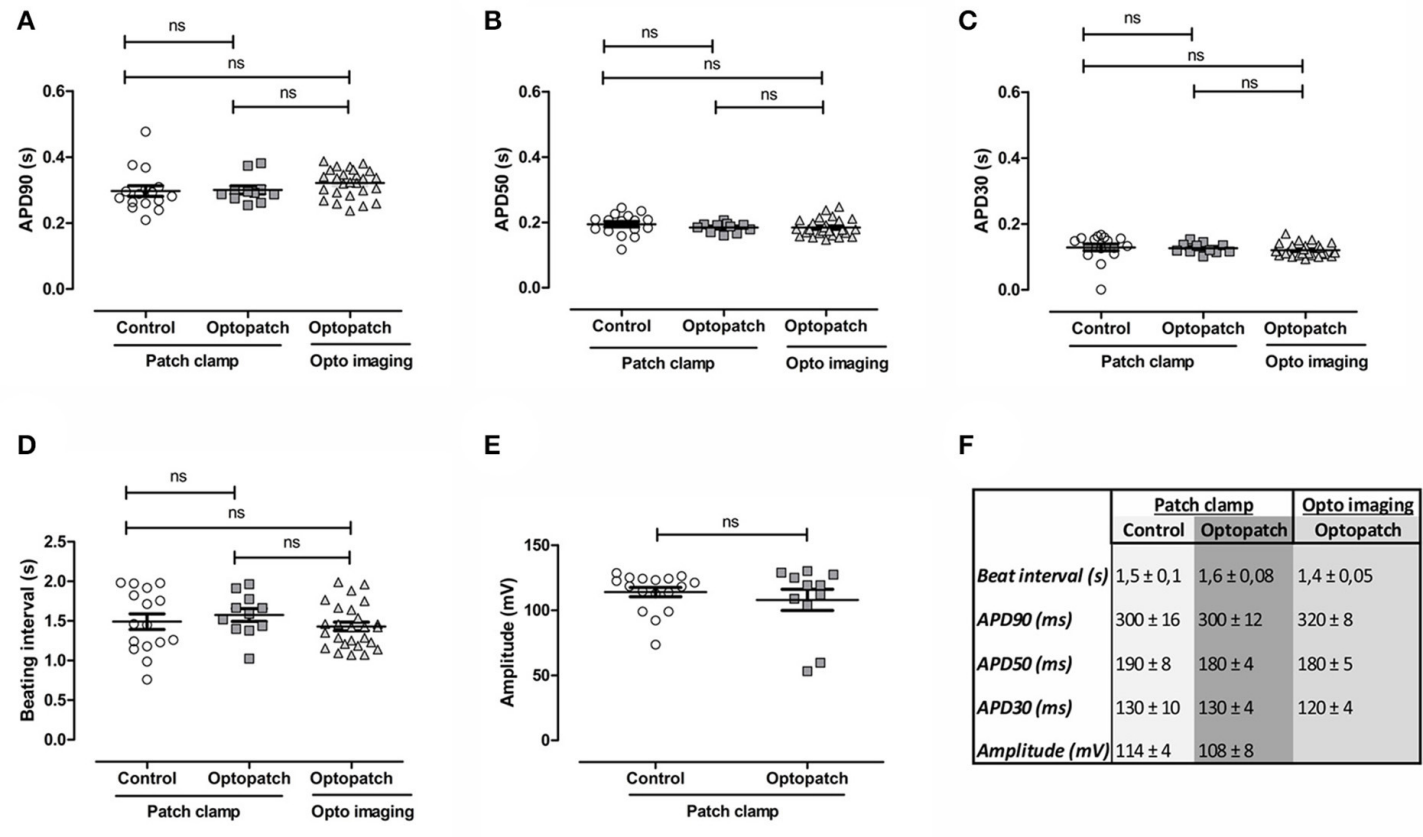
Validation of action potential parameters of virally non-transduced (control) and Optopatch-transduced hiPSC-derived cardiomyocytes with patch clamp electrophysiology and optogenetic imaging. Action potential parameters of Optopatch-transduced cells were measured with patch clamp electrophysiology and optogenetic (opto) imaging and the results compared against non-transduced control cells measured with patch clamp. **(A)** Action potential duration (APD) at 90% repolarization, **(B)** at 50% repolarization, and **(C)** at 30% repolarization, as wells as **(D)** beating intervals and **(E)** peak amplitude. **(F)** Summary of parameters measured in **(A–E)**, averages and S.E.M, control; *n* = 16, Optopatch measured with patch clamp; *n* = 11, Optopatch measured with optogenetic imaging; *n* = 26.

To validate the effect of lentiviral transduction of hiPSC-CMs with the optogenetic construct Optopatch (Hochbaum et al., 2014), we measured AP parameters with patch clamp electrophysiology and optogenetic imaging and compared the results against non-transduced control cells (Figure 1). There were no significant changes in the APD at 90% repolarization (Figure 1A), at 50% repolarization (Figure 1B), at 30% repolarization (Figure 1C), nor in beating rates (Figure 1D) between non-transduced cells and Optopatch-transduced cells. Nor were there any significant changes between the two methods of measurement: patch clamp electrophysiology and optogenetic imaging. The same stemmed for peak amplitude (Figure 1E), although this could only be compared using patch clamp electrophysiology, as the measured parameter for patch clamp (mV) is not measured by optogenetic imaging. A summary for the numerical AP parameters measured on non-transduced and Optopatch-transduced cells in Figures 1A–E is outlined in Figure 1F. Pearson correlations for the measured APD parameters were 0.99 (*p* < 0.001) both for non-transduced against Optopatch-transduced cells, as well as for correlations between APD parameters measured by patch clamp and optogenetic imaging. Thus, we propose that inserting the Optopatch construct into cardiomyocytes does not affect the cardiomyocyte's electrophysiological properties. To determine the amount of viral particles for delivery of the constructs to the cells, initial optimizations were performed with four different lentiviral concentrations of Optopatch, in which the lowest concentration yielded a monolayer of cells that did not pace at elevated frequencies and the highest concentration resulted in some cell death, therefore the second highest concentration of virus was used. However, screened by an absorbance-based LDH cytotoxicity method, this toxicological measurement revealed no significant cytotoxicity or cytolysis in any of the used lentivirus concentrations compared to non-transduced Cor.4U® cells (Supplementary Figure 1).

**Figure 2:**
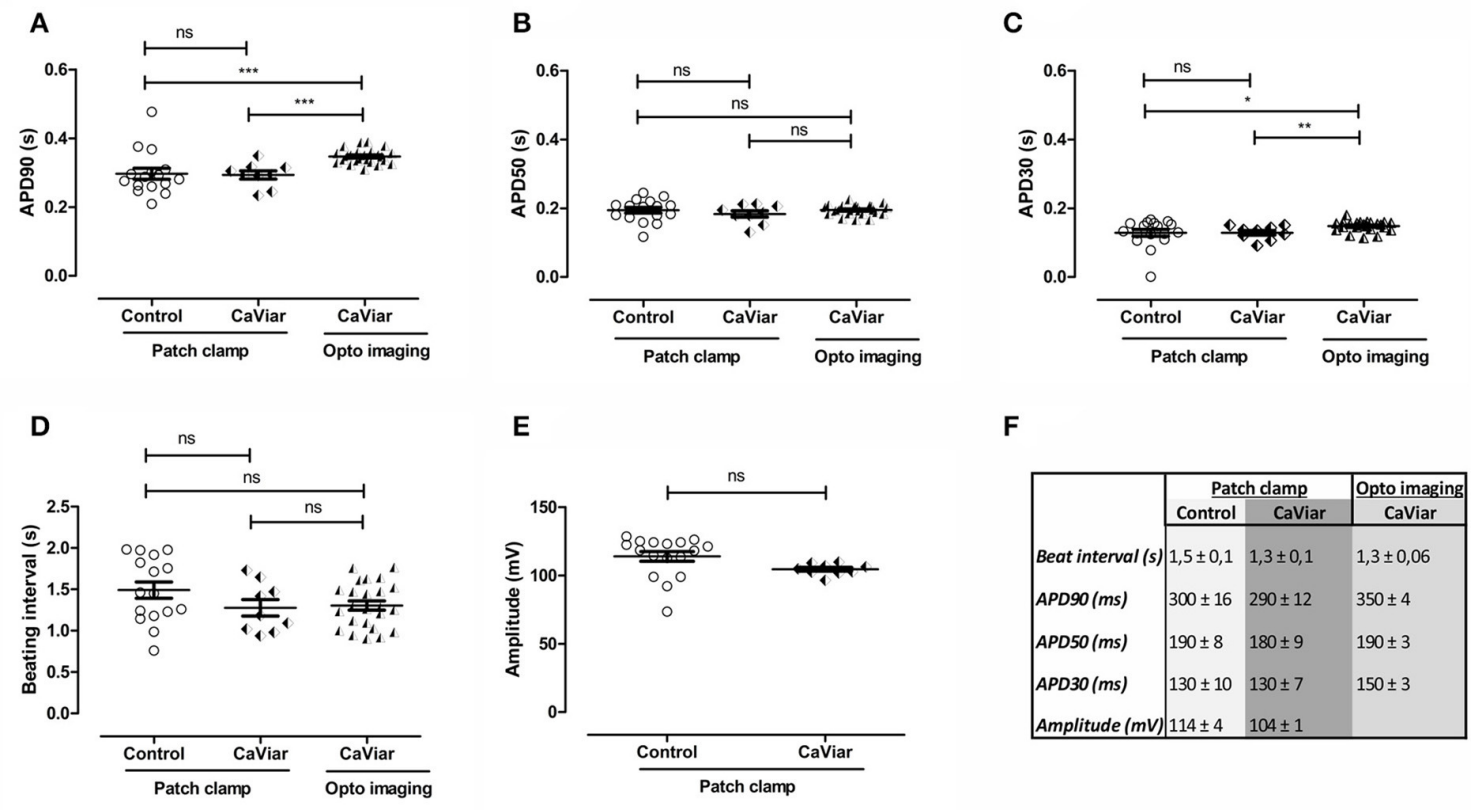
Validation of action potential parameters of virally non-transduced (control) and CaViar-transduced hiPSC-derived cardiomyocytes with patch clamp electrophysiology and optogenetic imaging. Action potential parameters of CaViar-transduced cells were measured with patch clamp electrophysiology and optogenetic (opto) imaging and the results compared against non-transduced control cells measured with patch clamp. **(A)** Action potential duration (APD) at 90% repolarization, **(B)** at 50% repolarization, and **(C)** at 30% repolarization, as well as **(D)** beating intervals and **(E)** peak amplitude. **(F)** Summary of parameters measured in **(A–E)**, averages and S.E.M, control; *n* = 16, CaViar measured with patch clamp; *n* = 9, CaViar measured with optogenetic imaging; *n* = 24. ^*^p < 0.05, ^**^p < 0.01, ^***^p < 0.001.

The optogenetic construct CaViar (Hou et al., 2014) holds the potential to measure changes in intracellular calcium in addition to AP dynamics and we therefore additionally validated the effect of transducing hiPSC-CMs with CaViar, and measured AP parameters (Figure 2) similarly as for the Optopatch construct. There were no significant changes in APD90 (Figure 2A), APD50 (Figure 2B), APD30 (Figure 2C), beating rates (Figure 2D) nor in amplitude (Figure 2E) between non-transduced cells and CaViar-transduced cells, measured by patch clamp electrophysiology. Pearson correlation values for the measured APD parameters were accordingly 0.99 (*p* < 0.001). This indicated that inserting the CaViar construct into hiPSC-CMs does not affect the electrophysiology of the cardiomyocyte. Neither was there any toxicity in any of the used concentrations of CaViar in toxicological measurement. However, when comparing APD parameters for the CaViar construct acquired with optogenetic imaging against those acquired with patch clamp electrophysiology, modest, but still statistically significant changes for APD90 and APD30 were seen. Thus, e.g., APD90 measured by patch clamp (300 ± 16 ms) and optogenetic imaging (350 ± 4 ms) exhibited a statistical difference, whereas APD50 did not. However, correlations values for all measured APD-values were still 0.98 (*p* < 0.001). A summary for the numerical AP parameters measured on non-transduced and CaViar-transduced cells in Figures 2A–E is outlined in Figure 2F.

### Simultaneous measurement of action potential, calcium transients, and contractile motion: signal characterization and timings

**Figure 3:**
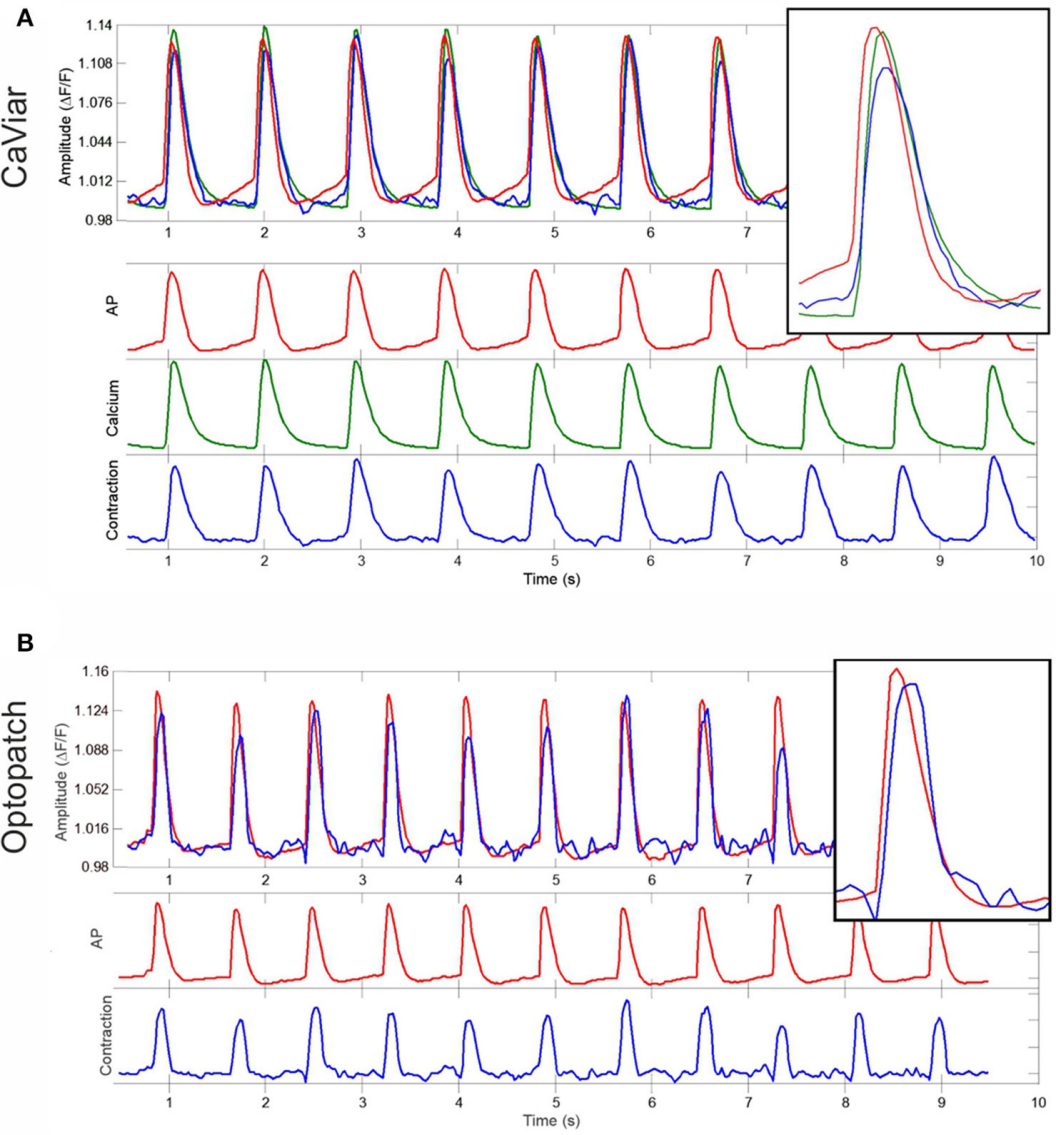
Simultaneous measurements of APD, calcium transients and cell contractility. Representative signals from **(A)** a CaViar-transduced cardiomyocyte recording showing the action potential (red), calcium transient (green), and contraction (blue) simultaneously, and from **(B)** an Optopatch-transduced cardiomyocyte recording showing the action potential (red) and contraction (blue), measured over time (s). Amplitudes describe changes in calcium **(A)** and action potential **(B)**. The insets are close-ups of a single peak.

Action potentials, calcium transients, and contractions were measured from CaViar and Optopatch recordings. Representative signals from a CaViar recording are shown in Figure 3A, and from an Optopatch recording in Figure 3B. The CaViar measurements displays the AP (red) preceding the calcium transient (green), which is then followed by a contraction (blue). Contraction ended rapidly after reaching a peak (slope coefficient −0.0108). The calcium transient curve showed very similar kinetics (slope coefficient −0.0114). The timing of peaks (Table 1) for both constructs well adheres to cellular physiology. There was ~30 ms interval between the AP and calcium peaks, and a 10 ms interval between the calcium and contraction peaks, thus a total of 40 ms between the AP and contraction peaks in CaViar measurements. For Optopatch measurements, the same value was 50 ms, albeit with a 30 ms variance indicating a close similarity to the AP-contraction dynamics of the two constructs. The Optopatch construct does not allow for calcium measurements and therefore the AP-Calcium interval could not be calculated. When measuring the directional velocities, contraction time was measured to be 180 ms in CaViar measurements and 180 ms in Optopatch measurements. Relaxation times were 270 and 230 ms, respectively. The difference was not statistically significant in a two-sample *t*-test.

**Table 1:** Simultaneous measurement of CaViar and Optopatch peak intervals and contraction time parameters.

	**AP-calcium peak interval (ms)**	**AP-contraction peak interval (ms)**	**Contractile time (ms)**	**Relaxation time (ms)**
CaViar	30 ± 20	40 ± 20	180 ± 30	270 ± 70
Optopatch	n.a.	50 ± 30	180 ± 30	230 ± 50

*The measures illustrate mean ± standard deviation (ms), (n = 10), n.a., not applicable; AP, action potential*.

The measured signals were further characterized by peak width parameters at 90, 50, and 30 signal amplitude levels. The results are shown in Table 2. None of the differences were statistically significant indicating very similar characteristics of the two constructs, CaViar and Optopatch. Linear correlations were calculated for the characterization parameters. The results were −0.14 for CD90/CTD90, 0.18 for CD50/CTD50, and 0.48 for CD30/CTD30.

**Table 2:** Characterization of calcium transient and contraction signals measured using CaViar and Optopatch.

	**CD90 (ms)**	**CD50 (ms)**	**CD30 (ms)**	**CTD90 (ms)**	**CTD50 (ms)**	**CTD30 (ms)**
CaViar	290 ± 60	170 ± 30	130 ± 30	470 ± 60	210 ± 20	140 ± 20
Optopatch	250 ± 40	170 ± 30	140 ± 30	n.a.	n.a.	n.a.

*CTD, calcium transient duration; CD, contraction duration. The measures illustrate mean ± standard deviation (ms), (n = 10), n.a., not applicable*.

### Optogenetic and patch clamp measurements show dose-dependent APD prolongation and early afterdepolarizations upon exposure to the hERG potassium channel blocker E-4031

**Figure 4:**
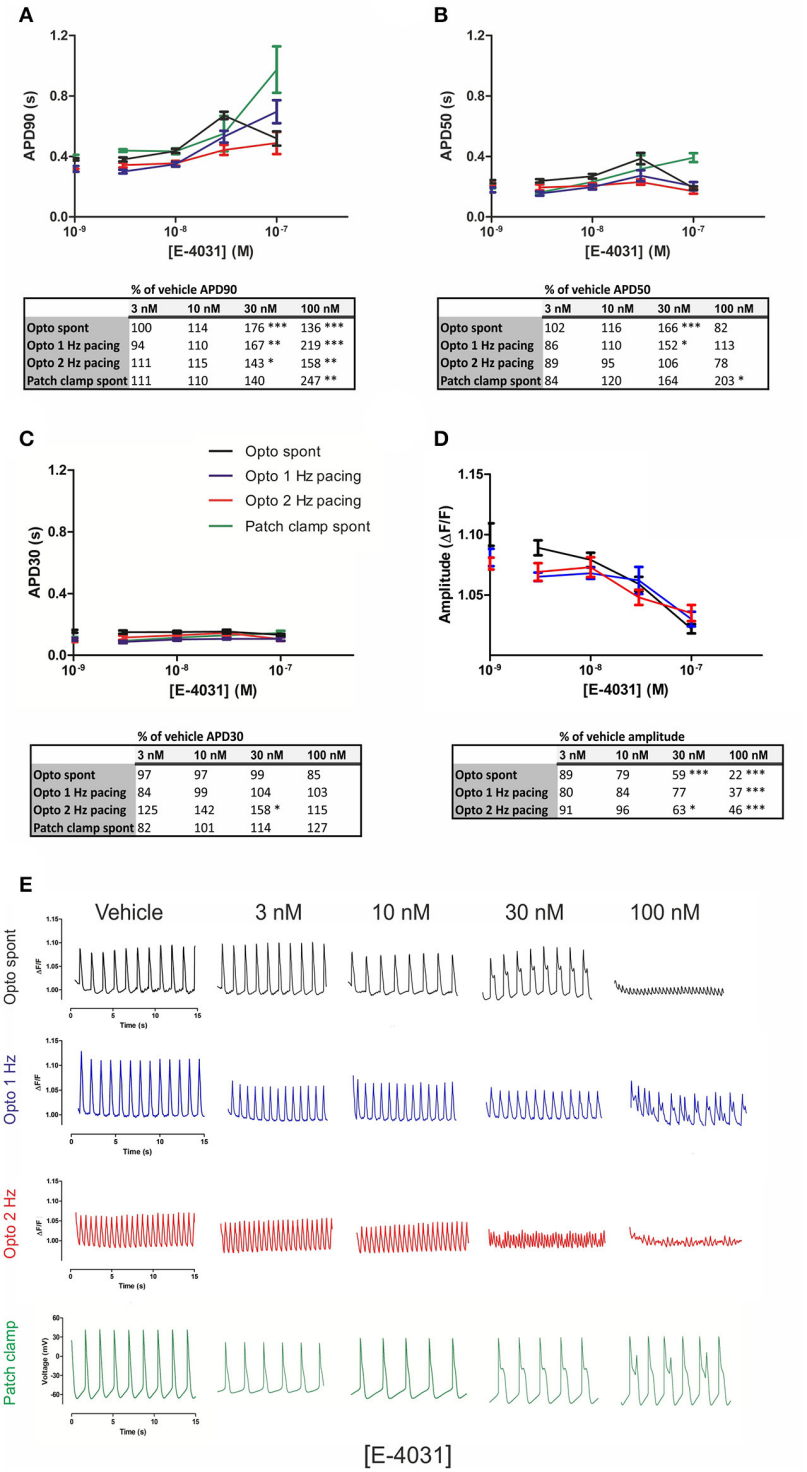
Optogenetic and patch clamp measurements of APD parameters under increasing doses of the hERG channel inhibitor E-4031. Quantification of the electrophysiological parameters **(A)** APD90, **(B)** APD50, **(C)** APD30, and **(D)** AP amplitude as a function of E-4031 concentrations, at spontaneous beating (black trace) rate and under 1 Hz (blue) and 2 Hz (red) pacing rates in the optogenetic imaging. Patch clamp measurements are represented as a green trace in **(A–C)**. Dots on y axis represent vehicle. Results are means ± S.E.M. from *n* = 14–20 (opto measurements) and *n* = 3–5 (patch clamp measurements). The table summarizes the percent change of the drug-induced response in comparison to vehicle and indicates the statistical significance, where ^*^p < 0.05, ^**^p < 0.01, ^***^p < 0.001. **(E)** Representative traces of alterations in the optical AP waveform induced by the indicated concentrations of the hERG channel inhibitor, E-4031 at spontaneous beating and under 1 and 2 Hz pacing rates, as well as action potential traces measured with patch clamp.

Many compounds have failed early on in drug development due to block of the hERG potassium channel, and we therefore assessed whether optogenetic measurements can show proarrhythmia events or early afterdepolarizations (EADs), which are known effects of hERG channel block. We utilized the potent and selective hERG blocker E-4031, as it commonly has been used as a positive control. Cumulating doses of E-4031 (3–100 nM) were applied to Optopatch-transduced hiPSC-CMs, and drug effects were measured by optogenetic imaging and by patch clamp. In optogenetic measurements, both spontaneous and optically paced beating rates (1 and 2 Hz) were screened, whereas patch clamp measurements only enabled recordings at spontaneous beating rates. Cumulating doses of E-4031 produced prolonged APDs dose-dependently in both methods (Figure 4).

In optogenetic measurements, averaged APDs from three dishes showed that APD90 at 30 nM E-4031 concentration was prolonged to 176% over vehicle APD90 (spontaneous beating), 167% under 1 Hz pacing and 143% at 2 Hz, with EADs evident at 30 nM under spontaneous and 1 Hz beat rates. APD90 was further increased to 219% over vehicle at 100 nM for 1 Hz and to 158% at 2 Hz. At 100 nM E-4031, APD90 decreased to 136% over vehicle under spontaneous beating which represented an average of the different behaviors seen; either very prolonged APDs with EADs or a decrease in peak amplitude with an increase in frequency, finally followed by drug-induced quiescence until beating stopped (Figures 4A,E). Similarly, in patch clamp measurements, the prolongation of APD90 (140%) was accompanied by EADs at 30 nM E-4031. APD90 further increased to 247% at 100 nM. In optogenetic measurements, a significant (*p* < 0.001) dose-dependent decrease in peak amplitude was evident at both spontaneous (22% over vehicle), 1 Hz (37%) and 2 Hz (46%) at 100 nM of E-4031 (Figure 4D). Representative traces for each drug concentration are shown in Figure 4E.

Contractile measurements revealed a dose-dependent (E-4031), non-significant prolongation of CD90 up to 10 nM (Table 3). EADs were detected as small twitches from 30 nM onwards after contraction and initial relaxation had occurred. E-4031 dose-dependently increased total relaxation time, being significant at 30 nM (*p* < 0.05). Motion magnitude decreased dose-dependently, reaching significance at 30 nM (*p* < 0.05). At 30–100 nM level, some image sequences could not be analyzed due to the motion reaching levels undetectable by the method.

**Table 3:** The effect of cumulating doses of E-4031 on CD90, relaxation time and motion magnitude in spontaneously beating Optopatch-transduced hiPSC-CMs.

**E-4031 (nM)**	**CD90 (ms)**	**Relaxation time (ms)**	**Change in motion magnitude (%)**
0 (vehicle)	220 ± 50	180 ± 50	100
3	230 ± 50	200 ± 50	99
10	250 ± 70	220 ± 80	71
30	180 ± 60^*^	270 ± 170^*^	59^*^
100	190 ± 90	280 ± 170^*^	22^*^

*The measures illustrate mean ± standard deviation (ms) in CD90 and relaxation time and percentage change in motion magnitude, (n = 18, 17, 20, 21 and 16, for vehicle, 3, 10, 30 and 100 nM, respectively)*.

* *p < 0.05*.

### The I_Ks_ blocker JNJ-303 prolongs APD slightly and decreases peak amplitude dose-dependently, under both spontaneous and elevated beating rates

**Figure 5:**
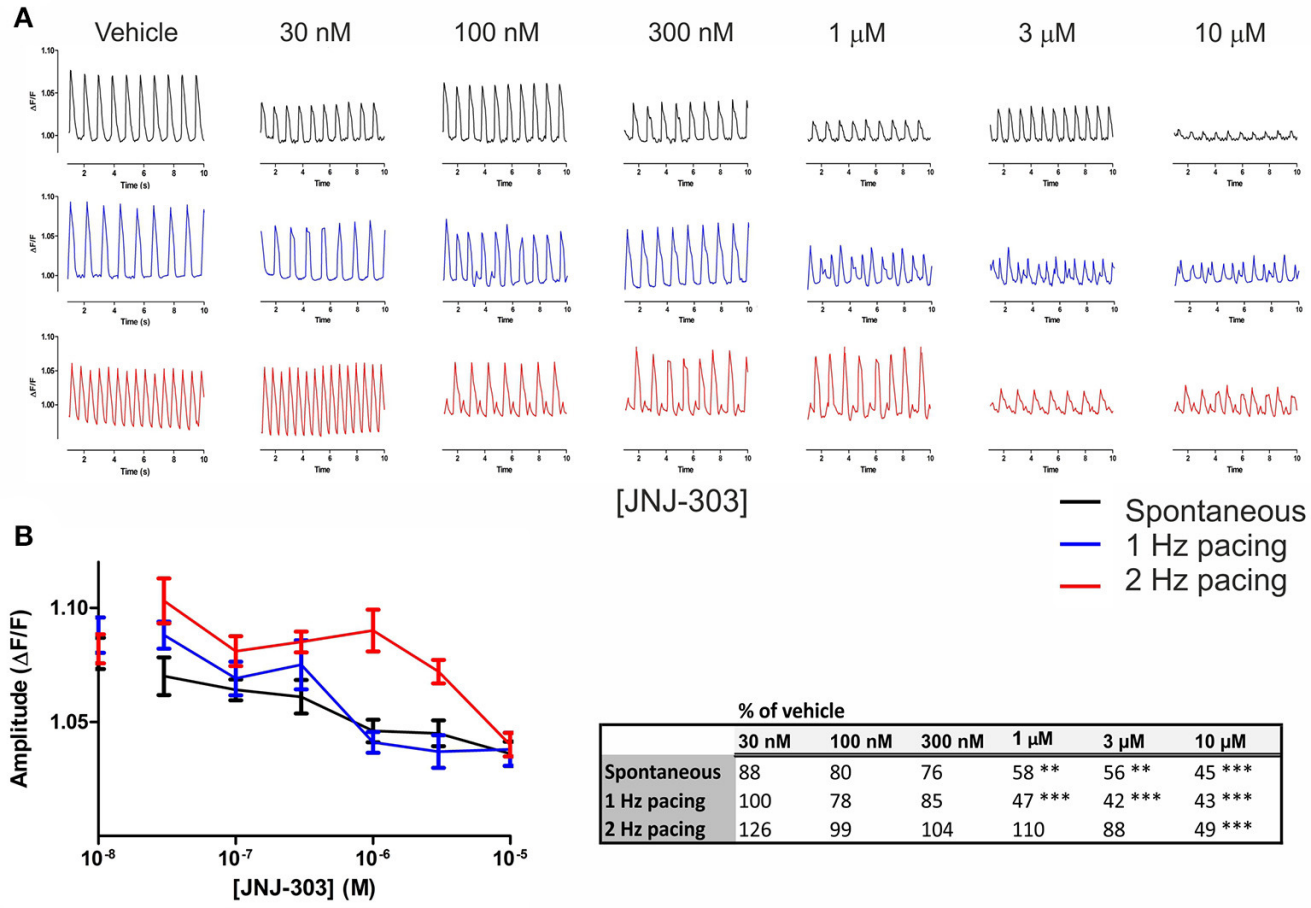
Optogenetic measurements of APD parameters under increasing doses of the I_Ks_ blocker JNJ-303. **(A)** Representative traces of alterations in the AP waveform induced by the indicated concentrations of the I_Ks_ blocker JNJ-303 at spontaneous beating (black) and under 1 (blue) and 2 Hz (red) pacing rates. **(B)** AP amplitude as a function of JNJ-303 concentrations, at the spontaneous beating rate and under 1 and 2 Hz pacing rates. Dots on y axis represent vehicle. Results are means ± S.E.M. from *n* = 14–25. The table summarizes the percent change in amplitude of the drug-induced response in comparison to vehicle and indicates the statistical significance, where ^**^p < 0.01, ^***^p < 0.001.

Cumulating doses of JNJ-303 (0.03–10 μM) were applied to hiPSC-CMs, and drug effects were measured at both spontaneous and 1–2 Hz paced beating rates (Figure 5). Prolongation of APD90 was seen at 100–300 nM JNJ-303 under spontaneous beat rates, and at 30–100 nM JNJ-303 under paced frequencies (Figure 5A). The prolongations were however statistically non-significant. A significant dose-dependent decrease in peak amplitude was evident already at 1 μM (58% of vehicle), decreasing to 45% at 10 μM under spontaneous beating. A similar decrease in the peak amplitude was also seen under 1 Hz pacing, whereas only 10 μM exhibited a significant decrease in peak amplitude (49% of vehicle) under 2 Hz pacing (Figure 5B). The cells stopped responding to pacing frequencies already at 100 nM of JNJ-303, but AP recordings were still continued under light stimulation at indicated pacing rates.

## Discussion

The present study was conducted to evaluate the optogenetic electrophysiology tools against the gold standard, patch clamp electrophysiology. Moreover, we evaluated how well-optogenetic tools and contractile motion measurements can predict proarrhythmia events and delayed repolarization (EADs) in cardiac drug safety screens.

Block of the potassium channel hERG plays a critical role in defining ventricular repolarization, however mechanistic and translational studies demonstrate that block of I_Kr_ alone is not highly specific for predicting either delayed repolarization or clinical proarrhythmia events (Gintant et al., 2016). Indeed, several drugs such as verapamil and ranolazine are potent hERG blockers, but are not associated with either QT prolongation or risk of TdP (Chouabe et al., 1998; Schram et al., 2004). It has been estimated that 60% of new molecular entities developed as potential therapeutic targets test positive in hERG blocking assays and are thus abandoned early on in the development pipeline (Gintant et al., 2016). However, these hERG-expressing immortalized cell-based assays do not represent the highly differentiated human cardiac myocyte. The technology of generating hiPSC-cardiomyocytes holds great potential for preclinical cardiac efficacy and safety screens (Grskovic et al., 2011; Matsa et al., 2014). These cells are somewhat immature, and phenotypically more close to an embryonic myocyte than adult, reflected in their less-negative resting potential, reduced upstroke velocities and spontaneous automaticity. In spite of this, hiPSC-CMs have been shown to respond in a highly predictable manner to over 40 compounds that have a known pharmacological effect on the human heart (Fermini et al., 2016). In addition, hiPSC technology enables the generation of cardiomyocytes from patients with e.g., congenital long-QT syndrome. Drug responses and toxicity measurements adapted on these cells could thus bring toxicity screens to a deeper level, stratifying patient responses and reducing late-stage clinical failures (Grskovic et al., 2011; Matsa et al., 2014). To overcome the shortcomings in current drug safety screens, the CIPA initiative proposes the investigation of drug effects on multiple human cardiac currents, tested in hiPSC-CMs.

Although hiPSC-CM transmembrane potential measured with patch clamp electrophysiology provide the most detailed characterization of electrophysiological effects on cellular repolarization, this technique is slow, technically demanding and very low-throughput (Gintant et al., 2016). Extracellular field potential recordings obtained through multielectrode arrays provide a means of adapting the assay to a high-throughput format, and communicates the rate of electrical activity and the timing of repolarization, but lack information regarding the morphological changes in the configuration of the AP and the actual end of repolarization. Voltage sensing optical platforms however offers significant advantage over these platforms (Chang Liao et al., 2015; Dempsey et al., 2016; Hortigon-Vinagre et al., 2016; Klimas et al., 2016), as they provide information on the whole AP waveform, which represent a readout of the integrated activity of multiple cardiac ion channels. Additionally, since the cardiotoxic effect of some drugs is evident only at elevated beat rates, insertion of genetically encoded actuators enables light-induced pacing of cells to higher beat frequencies. Combination of optogenetic pacing and voltage sensitive dyes has been reported (Park et al., 2014; Klimas et al., 2016), however the suitability of fluorescence dyes for long term incubations is questioned since fluorescent dyes can cause photodynamic damage to isolated cardiomyocytes under prolonged incubation (Schaffer et al., 1994) or potentially alter the electrophysiological properties of the cells (Novakova et al., 2008). HiPSC-CMs can be maintained in culture for a long time, and thus these cells *per se* (in contrast to primary animal myocytes) permit long-term experiments. Non-invasive optogenetic measurements on hiPSC-CMs transduced with genetically encoded optical actuators and sensors therefore enable *in vitro* evaluation of chronic drug effects and delayed cardiotoxicity, as reported in (Dempsey et al., 2016). Optogenetic electrophysiology assessment tools thus have the potential to improve sensitivity and specificity in the early detection of genuine cardiotoxicity risks, thereby reducing the likelihood of mistakenly discarding viable drug candidates and speeding the progression of worthy drugs into clinical trials (Dempsey et al., 2016; Gintant et al., 2016; Klimas et al., 2016).

We have evaluated hiPSC-CMs transduced with the commercially available constructs Optopatch and CaViar (Hochbaum et al., 2014; Hou et al., 2014) against non-transduced cells with patch clamp electrophysiology and shown that none of the measured AP parameters were affected. There were no significant differences in APD at 90, 50, or 30% repolarization, nor in peak amplitude or beat rates. The APD90 for Optopatch was 300 ± 12 ms measured with patch clamp and 320 ± 8 ms measured with optogenetic imaging, both of which compare well to the computational human ventricle APD90-value of 300 ms (O'Hara et al., 2011), as well as to reported APD90-values for ventricular-like hiPSC-CMs (312–320 ms) (Zhang et al., 2009; Lahti et al., 2012). The measured APD parameters were selected from recommendations by the CIPA initiative, and were calculated by a MATLAB-based software that was generated by us. When looking at how well the optogenetic imaging experiments compared to patch clamp measurements, we found no significant differences between the two techniques for the Optopatch construct (Figure 1), whereas for the CaViar construct the APD90- and APD30-values exhibited modest, but significant differences for APD parameters between the two techniques (Figure 2). We utilized the CaViar construct published by Hou et al. (2014) that contains the Arch(D95N) voltage indicator and not the QuasAr2 voltage indicator published by Dempsey et al. (2016), as this newer version of CaViar was not available from Addgene at the time of our experiments. Compared to QuasAr2, Arch(D95N) has been shown to exhibit slower responses to voltage transients (Gong et al., 2013), which might provide a possible explanation for the prolongation of the APD in the Caviar expressing cells measured with optogenetic imaging. The largest difference was seen at APD90, measured close to the base of the AP, where small changes in the AP waveform due to the slower kinetics of the Arch(D95N) can results in a broader AP waveform. The newer CaViar version based on the QuasAr2 voltage indicator might possibly alleviate this shortcoming.

In order to rule out most methodological restrictions related to the cellular material, we used only the well-documented, standardized and validated commercial Axiogenesis Cor.4U® hiPSC-CMs in our experimentations (typical confluent cell monolayer is illustrated in Supplementary Figure 2). Future experiments should be focused on ruling out the contribution of atrial hiPSC-CMs, as well as on comparing the results from Cor.4U® cells used in this study to ventricular-enriched hiPSC-CMs such as vCor.4U® cells. In the future, continued evaluation of the most optimal cell platform will provide great value in further validating the cellular system best compatible with the optogenetic electrophysiology tools. The experimental procedure for lentiviral delivery of the optogenetic constructs was quite time-consuming, as cells had to be kept at the viral core facility until RCV negative, which took 2–3 weeks, including washing and media exchange every day, as well as one passage of the cells which resulted in loss of cardiomyocytes. One of the bigger drawbacks of optogenetic imaging is the lack of ability to determine absolute values for the resting potential. However, the cardiac AP waveform is sensitive to resting membrane potential, and changes in APD are expected if compounds induce a shift in resting voltage (Dempsey et al., 2016). We can calculate percentile differences in amplitude peak height but not compare these to amplitudes measured by patch clamp, which yields accurate numerical values. The red voltage fluorescence trace photobleaches in the first few seconds of imaging, yielding a fluorescence trace with a steep descend. To solve this problem and to accurately measure AP parameters from curves, a normalization step was inserted in the automated processing of raw data by the cPot software. Dempsey et al. (2016) tested for photobleaching or phototoxicity arising from imaging of QuasAr2 in cardiomyocytes by measuring fluorescence during 500 s of continuous red laser illumination (500 mW/mm^2^) and showed a modest signal amplitude decrease of 12% during the acquisition with a small variability in the AP width, which was within the natural variation in spontaneously beating hiPSC-CMs.

Contractile and structural cardiotoxicity, seen with e.g., some kinase-targeted cancer drugs, represent another safety concern (Cheng and Force, 2010). Cellular electric impedance assays have been implemented with multielectrode assays for contractility measurements (Obergrussberger et al., 2016), but do not provide the spatial resolution to detect movements within the cell in detail, in contrast to the minimum quadratic difference method based on video motion tracking used here (Ahola et al., 2014, 2017). We aimed to evaluate whether optogenetic measurements of cardiomyocyte electrophysiology can be combined with an assay measuring the end point of the cardiomyocyte electrical activity, i.e., contraction and relaxation. To our knowledge this is the first study to combine video motion tracking with optogenetic measurement of APD and calcium transients (Figure 3, Table 1). The measurement setup provided a detailed view on the key components related to cardiomyocyte function, and revealed a physiological order of AP, calcium and contraction peaks (Bers, 2002). It widens the scope of studying drug effects as changes in any of the three signals can be quantified simultaneously. Motion analysis can also reveal information that is beyond the electrical properties and ion fluxes in cardiomyocytes, such as actual biomechanical timing and possible intracellular motion defects (Ahola et al., 2014). From the contractile motion point of view, Optopatch and CaViar appear to function similarly, as can be seen as the relatively similar values describing contraction-relaxation parameters between the two constructs (Tables 1, 2). None of the differences found were statistically significant (*p* < 0.05). The correlations between CD and CTD parameters indicated that a connection between the two can be found, but one cannot be directly deduced from the other. A very low correlation value was found in CD90, indicating high variance near baseline. This is not unexpected, as a prolongation in calcium transient near baseline does not equate to longer cell relaxation. The measured correlation values are lower than previously reported by Ahola et al. (2017) where the correlations were in the range of 0.6–0.7. However, the differences may be explained by a different calculation method of the measurement parameters in these two studies.

To assess how optogenetic measurement can reveal changes in cardiomyocyte repolarization we exposed the hiPSC-CMs to the hERG potassium channel blocker E-4031 (Figure 4). I_Kr_ inhibition by E-4031 prolonged APD in the late phase of repolarization consistent with the role of I_Kr_ in phase 3 of repolarization in the adult ventricular myocyte (Gintant, 2000). Cardiomyocytes showed cellular arrhythmias in response to cumulating doses of E-4031. 100 nM E-4031 has been shown to induce EADs in stem-cell derived cardiomyocytes (Peng et al., 2010). We, similarly to Obergrussberger et al. (2016) detected significant APD90 prolongation already at 30 nM under both spontaneous and paced beat rates in optogenetic measurements as well as under spontaneous beating measured by patch clamp. In both methods, the prolongation of APD under spontaneous beat rate was followed by EADs at 30–100 nM concentration of E-4031. In optogenetic imaging, however, exposure to 100 nM E-4031 more often resulted in a decrease in signal amplitude with an increase in frequency, followed by drug-induced quiescence. In patch clamp, the somewhat large variations in APD S.E.M.s were due to a small sample size in the labor-intensive patch clamp method. However, the two methods showed significant prolongation of APD90, as well as EADs detected at the same concentration (30 nM E-4031). Thus, we show that E-4031, which in clinical settings has been shown to cause the proarrhythmic event TdP, also when measured with optogenetic tools caused an increase in the APD and induced EADs. In contraction signals, E-4031 increased relaxation duration, decreased motion magnitude and caused EADs at 30 nM levels. The results suggest that contraction analysis can be a feasible tool in detecting drug responses in high-throughput applications. However, large sample sizes are required for definite conclusions as high concentrations applied to individual cells or small clusters may terminate the beating altogether, causing variances in measured parameters.

Repolarization of the cardiac AP is not only dependent on the hERG channel but on several ion channels including the I_Ks_, the second main potassium channel involved in ventricular repolarization, and thus the length of the QT interval. To assess whether optogenetics could detect activities of this channel, we applied JNJ-303, a potent blocker of I_Ks_, to hiPSC-CMs. Previous studies using this drug revealed no peculiar activity in standard hERG screens, but subsequently evoked unprovoked TdP *in vivo* in an anesthetized dog model (Towart et al., 2009). Our results (Figure 5) revealed a statistically non-significant, yet visible prolongation of the APD90 already at low concentrations of JNJ-303 under both spontaneous and paced rates, accompanied by possible delayed afterpolarizations (DADs). A significant decrease in signal amplitude starting from 1 μM JNJ-303, accompanied by an increase in frequency, which was followed by drug-induced quiescence and finally beating arrest, thus indicating blocking activity of cardiomyocyte repolarization. Additional experiments with a sodium channel blocker could have shed light on how inhibited depolarization could be measured by optogenetic imaging, though this has been already reported by Dempsey et al. (2016).

Finally, we have shown that optogenetics reliably can detect changes in the AP waveform, including APD prolongation, EADs, and drug-induced quiescence. Yet, we do not propose that optogenetic electrophysiology experiments completely replace comprehensive patch clamp electrophysiological assessments, but it allows for a faster prediction of successful and safe drug candidates in a high-throughput screening (HTS) format. Optogenetics could be utilized in the early stages of preclinical drug development and could thus extensively reduce cost for the pharmaceutical industry. Selected candidates taken further for clinical trials could then be studied in detail on a single cell level with patch clamp.

In conclusion, we have shown that optogenetic imaging allows for AP waveform recordings from a cardiomyocyte monolayer. Due to the non-invasiveness and non-toxicity of genetically encoded voltage sensors and actuators, chronic drug exposures are enabled. Furthermore, light-induced pacing of cells to elevated beat rates allows for arrhythmogenic sensitization. With the high throughput screening compatibility of hiPSC-CMs and the optogenetic technique, broader high content screens can be established with integrated contractility studies. Thus, optogenetic measurements provide an appealing alternative to electrophysiological screening of human cardiomyocyte responses for pharmacological efficacy and safety testing.

## Author contributions

SB, EO, TN, AA, ML, JH, EK, and EM: designed the research; SB, EO, and TN: collected the data; SB, EO, TN, and AA: analyzed the data; and SB, EO, and AA: wrote the manuscript. All authors reviewed the manuscript.

### Conflict of interest statement

The authors declare that the research was conducted in the absence of any commercial or financial relationships that could be construed as a potential conflict of interest.

## Supplementary material

Supplementary Figure 1 Virus-induced cytotoxicity (LDH release) measurements. **(A)** Optopatch-transduced hiPSC-CMs revealed no significant increase in cytotoxicity or cytolysis compared to control (Cor.4U® non-transduced cells, dotted line), measured at 24 h after lentiviral transduction. **(B)** Neither CaViar-transduced hiPSCs revealed any significant cytotoxicity over control cells. Results are means ± S.E.M. from *n* = 3. OD, optical density.

Supplementary Figure 2 Confluent monolayer of hiPSC-derived cardiomyocytes. Phase contrast microscopy image (20X) showing typical structural characteristics and cell density of confluent monolayer of Cor.4U® hiPSC-CMs cultured on Geltrex-coated glass-bottom dishes for optogenetic imaging. Scale bar = 100 μm.
