# Supplementary figures and images for: Evaluation of Optogenetic Electrophysiology Tools in Human Stem Cell-Derived Cardiomyocytes
Source: Front Physiol. 2017 Nov 2;8:884. doi: 10.3389/fphys.2017.00884 (PMC5673656; doi:10.3389/fphys.2017.00884)

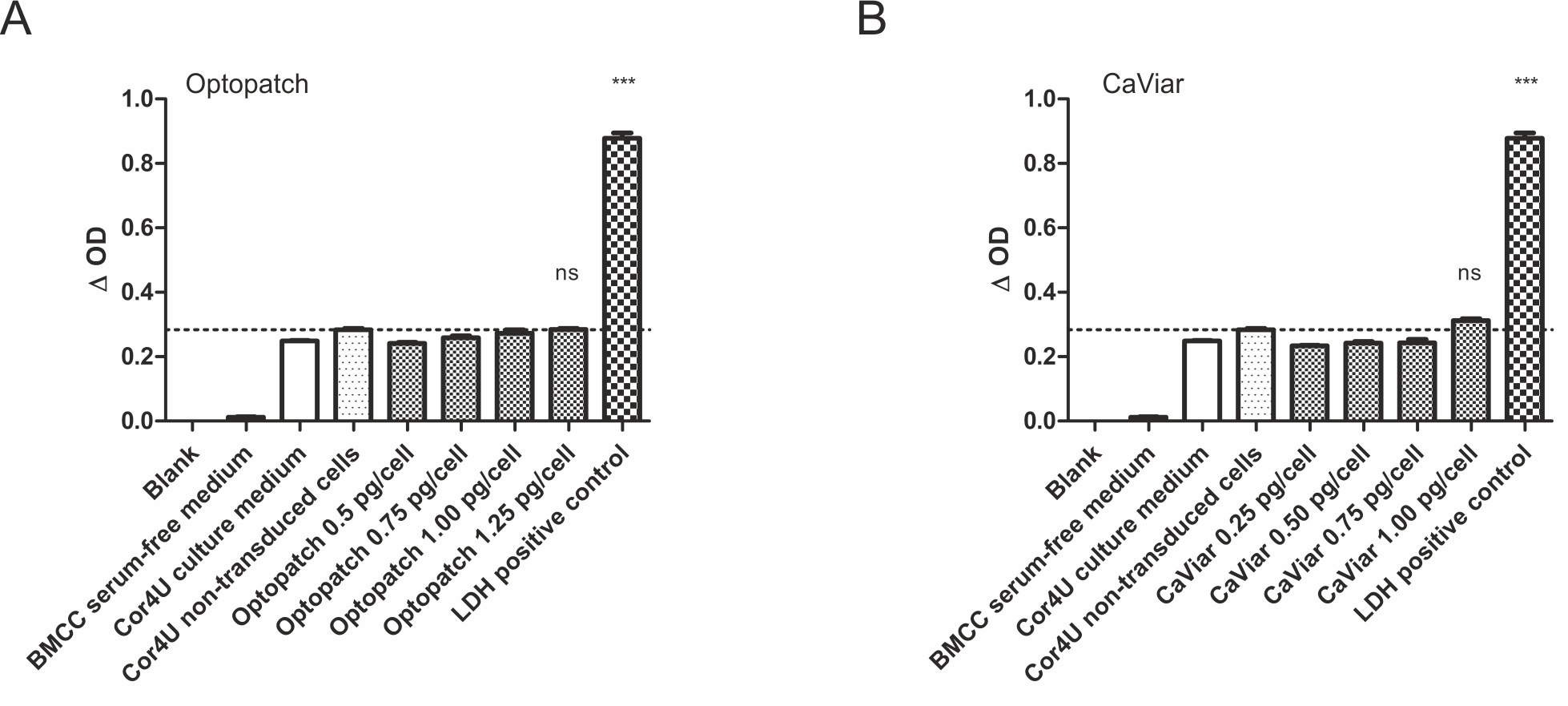

Supplement: Supplementary Figure 1 — Virus-induced cytotoxicity (LDH release) measurements. (A) Optopatch-transduced hiPSC-CMs revealed no significant increase in cytotoxicity or cytolysis compared to control (Cor.4U® non-transduced cells, dotted line), measured at 24 h after lentiviral transduction. (B) Neither CaViar-transduced hiPSCs revealed any significant cytotoxicity over control cells. Results are means ± S.E.M. from n = 3. OD, optical density. [file Image1.JPEG]

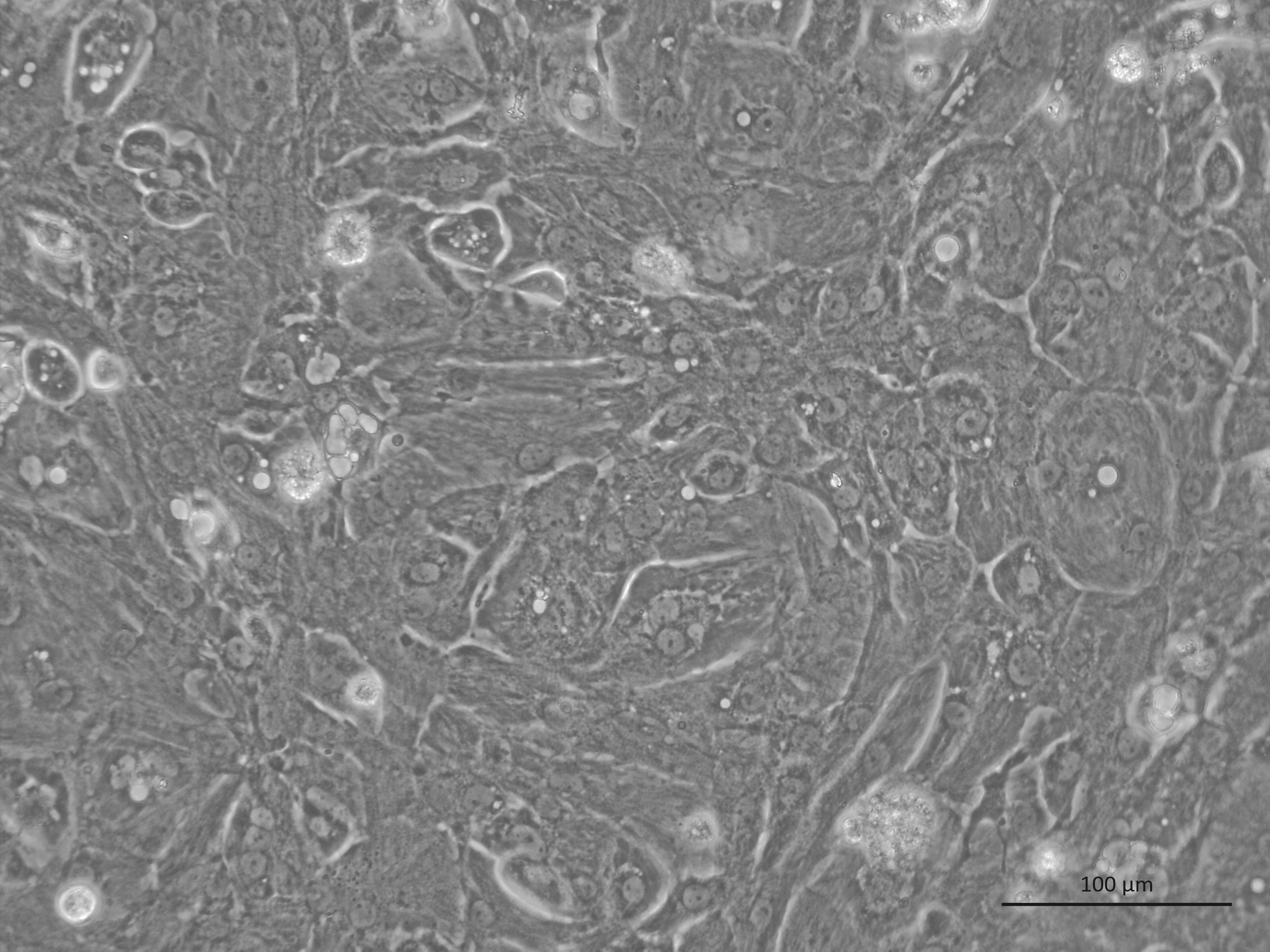

Supplement: Supplementary Figure 2 — Confluent monolayer of hiPSC-derived cardiomyocytes. Phase contrast microscopy image (20X) showing typical structural characteristics and cell density of confluent monolayer of Cor.4U® hiPSC-CMs cultured on Geltrex-coated glass-bottom dishes for optogenetic imaging. Scale bar = 100 μm. [file Image2.JPEG]
